# Targeting SHMTs and MTHFDs in cancer: attractive opportunity for anti-tumor strategy

**DOI:** 10.3389/fphar.2024.1335785

**Published:** 2024-02-20

**Authors:** Xue Zhang, Zhenhua Wang

**Affiliations:** ^1^ The VIP Department, School and Hospital of Stomatology, China Medical University, Shenyang, China; ^2^ Department of Physiology, School of Life Sciences, China Medical University, Shenyang, China

**Keywords:** metabolism, one carbon metabolism, metabolic enzyme, tumorigenesis, MTHFD family

## Abstract

One-carbon metabolism is a universal metabolic process that mediates the transfer of one-carbon units for purine and thymidine synthesis. One-carbon metabolism has been found to be dysregulated in various cancer types due to its role in production of purine and pyrimidine nucleotides, epigenetic program, and redox homeostasis. One-carbon metabolism is composed a network of one-carbon metabolic enzymes. Disturbing the expression and enzymatic activity of these one-carbon metabolic enzymes could lead to fluctuations of metabolites in the tumor microenvironment. Serine hydroxymethyltransferases (SHMTs) and methylenetetrahydrofolate dehydrogenases (MTHFDs) are gradually recognized as important one-carbon metabolic enzymes for regulating tumor initiation and development, representing potential therapeutic targets for anti-tumor strategies. In the review, we primarily focused on the role of SHMTs and MTHFDs in cancer. Several inhibitors targeting MTHFDs and SHMTs have exert its potential to decrease tumor burden and inhibit tumor proliferation, highlighting the potential of targeting one-carbon metabolic enzymes for anti-cancer strategies.

## Introduction

One-carbon metabolism, also known as folate metabolism, is a universal metabolic process that participates in the transfer of one-carbon units for purine and thymidine synthesis (Ducker and Rabinowitz, 2017). Altered one-carbon metabolism has been emerged as a critical feature of tumor cells, providing building blocks for nucleotide biosynthesis, epigenetic regulation and redox homeostasis (Newman and Maddocks, 2017). One-carbon units are required for the synthesis of purine and pyrimidine nucleotides, which are necessary for DNA and RNA synthesis. Due to the enhanced requirement of nucleotides for proliferation, tumor cells demand increased one-carbon units for nucleotide synthesis (Yang et al., 2021). The interplay between one-carbon metabolism and epigenomic program is also crucial for tumorigenesis. DNA methylation is an epigenetic mechanism that is essential for regulating gene transcription, and the aberrant DNA methylation changes is commonly observed in tumors (Friso et al., 2017) The epigenetic implications of one-carbon metabolism dysregulation in tumor cells are required further study. NADH and NADPH are important co-factors that provide electrons for redox reactions, which can be generated from one-carbon metabolism (Pan et al., 2021a). Herein, one-carbon metabolism is important for tumor initiation and progression.

Targeting the one-carbon metabolism provokes antifolate chemotherapeutic regimens to treat malignant tumors. Currently, the FDA-approved drugs targeting one-carbon metabolic enzymes includes methotrexate, pemetrexed, pralatrexate, trimetrexate, pyrimethamine and 5-Fluorouracil (Cuthbertson et al., 2021). Pemetrexed and 5-fluoruoracil, these two classic antifolate chemotherapeutics, are active in various solid tumors and hematological tumors. Pemetrexed inhibits several enzymes involved in the folate pathway including Dihydrofolate reductase (DHFR), thymidylate synthase (TYMS) and serine hydroxymethyltransferase (SHMT), while 5-fluoruoracil specifically targets TYMS. However, these drugs act non-selectively on normal tissues and tumor tissues, which can not only kill tumor cells to play anti-tumor role, but also rapidly leads to exhaustion of circulating myeloid and lympho-progenitor cells to impair the immune response. Therefore, future therapeutics should be more specifically targeting one-carbon metabolism in tumor cells by more selectively targeting individual one-carbon pathway enzymes. Some other one-carbon enzymes have been found to be selectively upregulated in multiple tumors, making these enzymes promising targets for the development of novel chemotherapeutic agents (Zhao et al., 2021).

**FIGURE 1:**
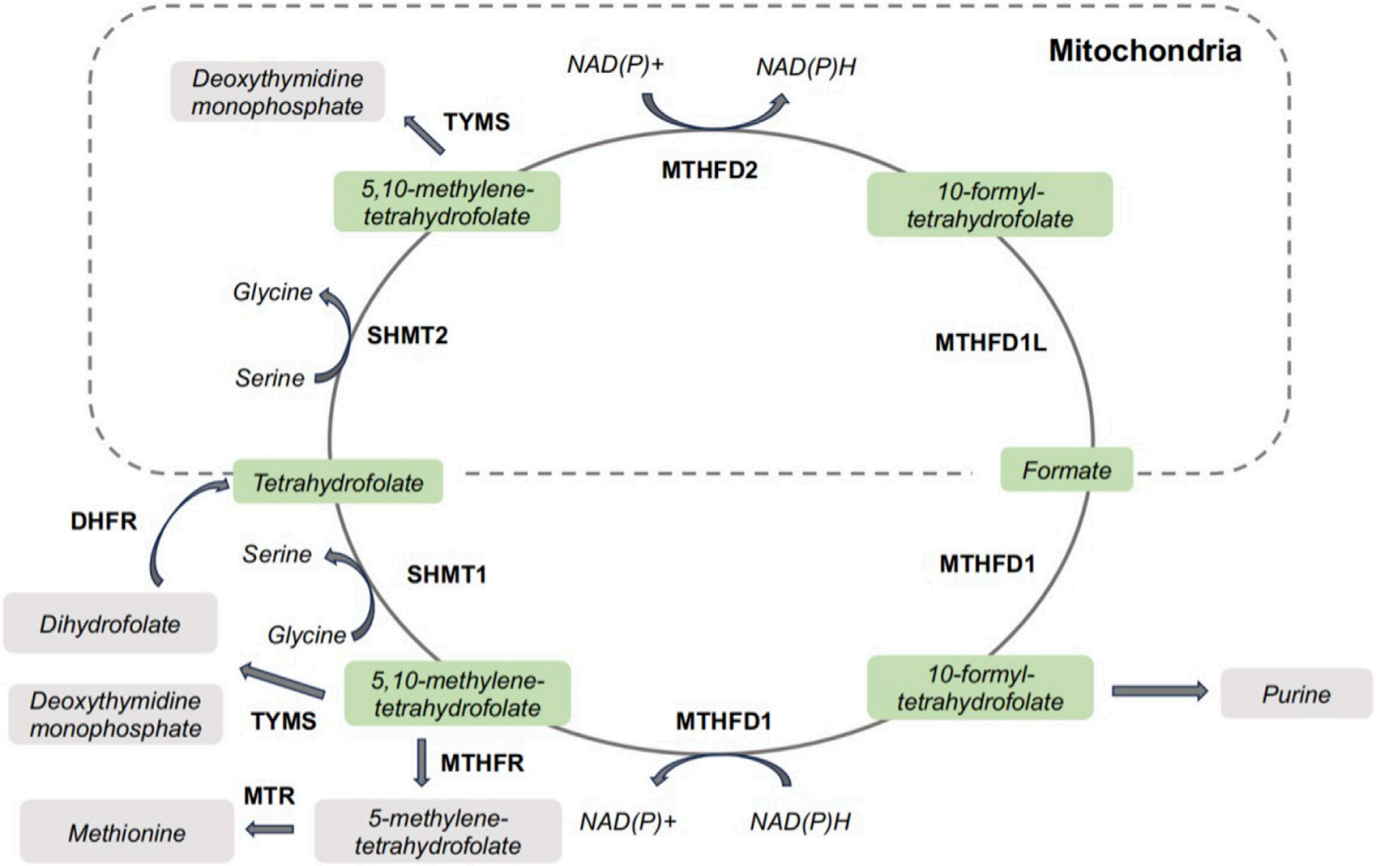
One-carbon metabolism in cells. DHFR, dihydrofolate reductase; MTHFD1, methylenetetrahydrofolate dehydrogenase/cyclohydrolase 1; MTHFD2, methylenetetrahydrofolate dehydrogenase/cyclohydrolase 2; MTR, methionine synthase; MTHFR, methylenetetrahydrofolate reductase; SHMT1, serine hydroxymethyltransferase 1; SHMT2, serine hydroxymethyltransferase 2; TYMS, thymidylate synthetase.

Generally, serine by uptake from extracellular environment or *de novo* biosynthesis is the main supply of the one-carbon units. Other amino acids, such as glycine, can also donate a one-carbon unit via glycine cleavage system. The uptake of extracellular serine is mediated by solute carrier family 1 member 4/5 (SLC1A4/SLC1A5), while serine biosynthesis begins with glycolytic intermediate 3-phosphoglycerate and underwent a series of reactions catalyzed by phosphoglycerate dehydrogenase (PHGDH), phosphoserine aminotransferase 1 (PSAT1), and phosphoserine phosphatase (PSPH) for *de novo* serine biosynthesis. Folate molecules function as carriers for 1C units, allowing them to be manipulated and assembled in support of metabolic processes. Once dihydrofolate reductase catalyzed the reduction of folate to tetrahydrofolate, SHMT1 could transfers a one-carbon unit from serine to from 5,10-methylene-tetrahydrofolate in cytoplasm. SHMT2, as the mitochondrial isoform of SHMTs, catalyzes the same enzymatic reaction to provide a one-carbon unit to form 5,10-methylene-tetrahydrofolate (Cuthbertson et al., 2021). Uniquely among the mitochondrial folate-related reactions, the oxidation of 5,10-methylene-tetrahydrofolate to 10-formyl-tetrahydrofolate can be catalyzed by two isozymes, MTHFD2 and MTHFD2L (Tibbetts and Appling, 2010). MTHFD1L catalyzes the conversion of 10-formyl-tetrahydrofolate to formate and transport formate out of the mitochondria. The illustration of one-carbon metabolism has been depicted in Figure 1. One-carbon pathways are compartmentalized in the cytosol, nucleus and mitochondria (Dekhne et al., 2020). Cytosol one-carbon metabolism has been exploited therapeutically for anti-tumor strategy, and antifolates targeting cytosolic one-carbon pathways are still primary chemotherapeutic regimens for anti-tumor strategy. In the one-carbon metabolism, glycine and serine could activate the mitochondrial enzymes SHMT2 and MTHFD2, which is critical for tumor cell survival (Cuthbertson et al., 2021). SHMTs and MTHFDs are not getting as much attention as other one carbon metabolic enzymes, like TYMS. Considering the potential of SHMTs and MTHFDs for developing anti-tumor strategies, we comprehensively summarized the role of SHMTs and MTHFDs in cancer initiation and progression. The clinical implications of SHMTs and MTHFDs are also discussed.

### Targeting MTHFDs in cancer

Members of MTHFD family are composed of MTHFD1, MTHFD1L, MTHFD2, and MTHFD2L. Recent studies have revealed that MTHFD1/2 have been identified as oncogenic enzymes upregulated in various tumors, which may highly participate in the initiation and progression of tumors. And it has been demonstrated that MTHFD1L/2L has limited effect in tumor initiation and progression. Herein, we primarily discussed the complex role of MTHFD1/2 in the anti-tumor immunity. The diverse role of MTHFD1/2 in different tumor types has also been summarized. The clinical implications of MTHFD1/2 in cancer have been outlined.

### Targeting MTHFD1 in cancer

Arginine methyltransferase 5 (PRMT5) mediates symmetric dimethylation of MTHFD1 on Residue-173. Elevated methylation of MTHFD1 could enhance its metabolic activity for the generation of NADPH, contributing to anoikis resistance and metastasis. Symmetric dimethylation of R173 was correlated with metastasis and prognosis of esophageal squamous cell carcinoma patients (Meng et al., 2022). During the metastatic process, melanoma cells exhibited elevated expression of one-carbon enzymes for the generation of NADPH (Piskounova et al., 2015). Inhibition of one-carbon pathway by MTHFD1 knockdown could attenuate distant organ metastasis of melanoma. In cholangiocarcinoma (CAA), MTHFD1 participated in the regulation of redox status, leading to chemoresistance to gemcitabine. Accordingly, anti-folate compound methotrexate targeting MTHFD1 could elevate ROS level, and the combination of gemcitabine with methotrexate could effectively suppress CAA growth (Pan et al., 2021b).

### Targeting MTHFD2 in cancer

The regulation of MTHFD2 is complex and dynamic. p53 could suppress MTHFD2 transcription, and MTHFD2 upregulation by p53 inactivation results in enhanced one-carbon metabolism, purine biosynthesis and tumor proliferation (Li et al, 2021a). MYC transcriptionally acts on MTHFD2, which is regulated by activating transcription factor 4 (ATF4) (Pikman et al., 2016; Pällmann et al., 2021; Gao et al., 2022). MicroRNAs also regulate MTHFD2 expression. miRNA-99a-3p has been found to be an upstream regulator directly inhibiting MTHFD2 expression in lung adenocarcinoma (Mo et al., 2022). In acute myeloid leukemia (AML), miR-92a could suppress cell proliferation by directly inhibiting MTHFD2 expression (Gu et al., 2017). The post-modification of MTHFD2 is complex and dynamic. Under folate stress, SIRT4 mediates the acetylation of MTHFD2 at K50 to regulate its protein stability, therefore driving tumor cell growth (Zhang et al., 2022). Sirtuin 3 (SIRT3), a key mitochondrial deacetylase, mediates the deacetylation of MTHFD2. MTHFD2 is hyperacetylated at lysine 88, which is the common acetylated site (Wan et al., 2020).

One-carbon metabolism is essential for T cell proliferation and function (Ron-Harel et al., 2016). Targeted screening in primary murine T cells has identified MTHFD2 as a key gene in inflammatory disorders. Given that one-carbon metabolism links various energetic sources and is therapeutically targetable, the role of MTHFD2 in CD4^+^ T effector (Teff), pathogenic T helper-17 (Th17) and regulatory T (Treg) cell subsets has been investigated. It has been demonstrated that MTHFD2 functions as a metabolic checkpoint determining T cell differentiation. In Th17 cells, MTHFD2 deficiency promotes aberrant upregulation of FoxP3 and suppressive capacity. MTHFD2 deficiency also induces Treg cell differentiation. These effects are correlated with impaired *de novo* purine synthesis, 5-aminoimidazole carboxamide ribonucleotide (AICAR) accumulation, guanine depletion, suppressed mTORC1 signaling, enhanced oxidative phosphorylation, and decreased methylation. Herein, MTHFD2 functions as a metabolic checkpoint balancing Th17 and Treg cells, highlighting MTHFD2 as a potential target for inflammatory disease (Sugiura et al., 2022). One-carbon metabolic network is also essential for Teff responses in tumors (Kurniawan et al., 2020). Functional screening has identified MTHFD2 as a driver metabolic gene contribute to resistance against Teff cells. It has been reported that MTHFD2 promotes basal and IFN-γ-induced PD-L1 upregulation, which is important for tumorigenesis. IFN-γ could induce MTHFD2 expression by activating AKT-mTORC1 signaling. MTHFD2 enhances PD-L1-induced resistance to tumor immunity through the one-carbon cycle-HBP pathway and the UDP-GlcNAc-O-GlcNAcylation-MYC-PD-L1 signaling pathway (Shang et al., 2021). These findings indicate the role of MTHFD2 in immune evasion, which may provoke further investigations on tumor immunotherapy.

One-carbon metabolism has been demonstrated to be highly correlated with initiation and development of colorectal cancer (CRC). Various one-carbon metabolic enzymes, including SHMT2 and MTHFD2, are significantly upregulated in CRC tissues than non-tumor tissues. Notably, patients with high expression of SHMT2 and MTHFD2 exhibit lower survival rates than patients with low expression of SHMT2 and MTHFD2 (Miyo et al., 2017). In CRC cells, MTHFD2 has been identified as a new deacetylation substrate of SIRT3. SIRT3 could deacetylate MTHFD2 at lysine 88 to alter its enzymatic activity. Furthermore, deacetylated MTHFD2 could enhance its enzymatic activity and regulate cellular levels of NADPH. Notably, cisplatin could reduce SIRT3 expression and ultimately increase MTHFD2 acetylation, which leads to reduced NADPH levels and increased ROS levels. This cisplatin-SIRT3-MTHFD2 axis in ROS production suggests MTHFD2 K88 acetylation as a potential target for CRC treatment (Wan et al., 2020). It has been found that protein levels of SIRT3 are negatively correlated with K88 MTHFD2 acetylation in CRC tissues, indicating MTHFD2 or SIRT3 inhibition may be promising for CRC treatment. Under oxidative stress, MTHFD2 maintains the level of NADPH. MTHFD2-mediated ROS balance could rescue CRC cells from demands of NADPH production, therefore promoting tumor progression. The folate analog LY345899 as MTHFD2 inhibitor exerts anti-tumor effect in CRC and provokes further investigation for anti-tumor treatment of CRC (Ju et al., 2019). In colon cancer cells, MTHFD2 promotes non-homologous end joining in response to DNA damage through the formation of complex with PARP3 to enhance its ribosylation. MTHFD2 silencing could restrain p53-deleted cell proliferation and sensitize tumor cells to chemotherapeutic regimens, suggesting potential of MTHFD2 inhibition for the treatment of p53-deleted tumors (Li et al., 2021b). Silencing the expression of MTHFD2 leads to impaired proliferation and migration of CRC cells, arrested in G0/G1-S phase, and increased apoptosis of CRC cells (Wei et al., 2019). In colon cancer cells, MTHFD2 has been confirmed to be the target gene of miR-33a-5p. miR-33a-5p could inhibit cell proliferation and migration of CRC by targeting MTHFD2 (Yan et al., 2019).

Castration-resistant prostate cancer (CRPC) occurs after androgen deprivation is an huge obstacle in developing anti-tumor strategies (Armstrong et al., 2019). Protein tyrosine phosphatase receptor type F polypeptide interacting protein alpha 4 (PPFIA4), localizing to mitochondria, could interact with MTHFD2 for one-carbon metabolism. Androgen deprivation could promote PPFIA4 translocation into mitochondria and enhance the interaction between PPFIA4 and MTHFD2, leading to increased tyrosine phosphorylated MTHFD2. Phosphorylated MTHFD2 increases the levels of NADPH, preventing androgen deprivation-induced mitochondrial dysfunction and promoting tumor growth. DS18561882, an MTHFD2 inhibitor, combined with enzalutamide (a potent androgen-receptor inhibitor) could suppress cell proliferation of CRPC (Zhao et al., 2022). Besides, MYC interacts with ATF4 to activate gene expression of one-carbon cycle in prostate cancer cells, including MTHFD2. MTHFD2 silencing could inhibit prostate cancer cell growth *in vitro* and patient-derived xenografts. Additionally, MTHFD2 inhibition via nanoliposomal siRNA could impair tumor growth *in vivo* models of prostate tumor (Pällmann et al., 2021).

MTHFD2 expression has been found to be highly upregulated in ovarian cancer than nontumoral samples (Cui, et al., 2022a). Ovarian cancer patients with high MTHFD2 expression have been found to be associated with lower survivals. MTHFD2 silencing could induce cell apoptosis and cell cycle arrest, impair ovarian tumor cell proliferation and metastasis. The inhibitory effect of MTHFD2 silencing on ovarian cancer progression may be resulted from inhibited expression and activity of cyclin B1/Cdc2 complex. MTHFD2 could promote tumor progression via STAT3-mediated epithelial-mesenchymal transition (Li et al., 2021a). In UQCR11-deficient ovarian cancer, MTHFD2 functions as a collateral lethal gene. MTHFD2 provides mitochondrial NAD+, and the UQCR11-MTHFD2 collateral lethality has been verified in mousemodels (Achreja et al., 2022).

AML is a heterogenous hematological malignancy of the stem cell precursors of the myeloid lineage (Löwenberg et al., 1999). Enzymatic function of MTHFD2 sustains rapid cell proliferation during early embryogenesis, while transforming to MTHFD2L in mature cells. In tumoral tissues, the re-activation of MTHFD2 indicates an isoform shift from MTHFD2L to MTHFD2 during tumor transformation. MTHFD2 silencing in AML cells could promote cell differentiation in primary AML blasts. In xenografts and mouse models, MTHFD2 silencing could decrease tumor burden and prolong survival. Functional genomic screening identified FLT3-ITD as a biomarker of response to MTHFD2 inhibition. Mechanistically, MYC could regulate the expression of MTHFD2, and MTHFD2 silencing inhibits the TCA cycle (Pikman et al., 2016). MTHFD2 has also implicated in DNA and replication stress. MTHFD2 inhibitors could retard replication fork and induce replication stress in AML cells. Mechanistically, MTHFD2 inhibitors could inhibit thymidine production resulting in DNA and replication stress. The interplay between MTHFD2-related tumor metabolism and replication stress that may be exploited therapeutically for cancer treatment (Bonagas et al., 2022).

Lung cancer is the leading cause of tumor-related deaths worldwide (Sung et al., 2021). MTHFD2 has been found to be upregulated in stage-dependent lung tumor tissues and lung tumor cell lines (Chan et al., 2020). MTHFD2 silencing could impair cell viability, transformation and self-renewal abilities of lung cancer cells. MTHFD2 silencing also reduces NADPH level and induce oxidative stress with increased ROS and cell apoptosis (Chan et al., 2020). The inhibitory effect of MTHFD2 silencing on tumorigenesis and stemness has been found to be correlated with purine depletion, which leads to the accumulation of AICAR-the final intermediate of the purine biosynthesis. Lung tumor cells with acquired resistance to the gefitinib exhibit enhanced stemness and upregulated MTHFD2 expression. MTHFD2 silencing or adding AICAR could reduce stemness and restore sensitivity to gefitinib in the gefitinib-resistant lung tumor cells (Nishimura et al., 2019). MTHFD2 overexpression in gefitinib-responsive lung tumor cells could confer gefitinib resistance. Thus, MTHFD2-induced one-carbon metabolism is important for tumor stemness and gefitinib resistance by reducing intracellular AICAR. Considering cancer stem cells rely on MTHFD2, MTHFD2 may be a target to eradicate stem cells and reduce recurrence.

In glioblastoma, MTHFD2 inhibition could activate the PERK/eIF2α axis, blocking translation and inducing apoptosis, which can be suppressed by a PERK inhibitor. Mechanistical study revealed that MTHFD2 is related to unfolded protein response via the post-transcriptional modification of chaperone protein GRP78. MTHFD2 mediates the progression of glioblastoma via unfolded protein response, indicating a novel link between one-carbon metabolism and stress response. Therefore, MTHFD2 is an attractive target for glioblastoma. (Zhu et al., 2022a). MTHFD2 also participated in cell cycle progression in bladder cancer. Nuclear MTHFD2 could activate CDK2 to promote the growth of bladder cancer by promoting cell cycle progression. (Liu et al., 2021a). In renal cell carcinoma, MTHFD2 get involved in regulating global N6-methyladenosine (m6A) methylation levels. Specifically, MTHFD2 induced m6A methylation of HIF-2α mRNA and enhanced translation of HIF-2α. Increased HIF-2α translation further promoted glycolysis to promote progression of renal cell carcinoma (Green et al., 2019).

### Clinical implications of MTHFD1/2 in cancer

An analysis of mRNA profiles of metabolic enzymes across 19 tumor types to explore metabolic enzymes that are differentially expressed indicates mitochondrial one-carbon metabolism as the highest scoring pathway, especially MTHFD2. The analysis of metabolic enzyme expression indicates an essential role of one-carbon metabolism and MTHFD2 in tumorigenesis and progression (Nilsson et al., 2014). MTHFD2 expression has been found to be dysregulated in multiple tumor types, especially solid and hematological tumors. MTHFD2 expression is correlated to clinicopathological features and clinical outcomes, implying the potential of MTHFD2 expression as a prognostic indicator in a disease-specific manner. MTHFD2 expression has been found to be upregulated in multiple tumor types (illustrated in Table 1).

**TABLE 1:** Expression patterns and clinical value of one-carbon metabolic enzymes in tumors.

Gene	Tumor type	Expression	Level	Clinical value	Reference
MTHFD1	Hepatocellular carcinoma	Upregulation	Protein	Shorter OS	Yu et al. (2019)
MTHFD2	Colorectal cancer	Upregulation	Protein	Shorter OS and DFS	Ju et al., 2019
					Miyo et al. (2017)
	Ovarian cancer	Upregulation	Protein mRNA	Shorter OS	Cui, et al. (2022b)
		Upregulation			
	Lung cancer	Upregulation	mRNA and protein	N/A	Shi et al. (2021)
	Glioblastoma	Upregulation	Protein	Advanced differentiation grade	Nishimura et al. (2019)
			mRNA	Advanced tumor stage	
	Bladder cancer	Upregulation	mRNA and protein	Poor prognosis	Zhu et al. (2022a)
SHMT1	Hepatocellular carcinoma	Downregulation	Protein	Shorter OS	Dou et al. (2019)
	Renal cell cancer	Downregulation	Protein mRNA	Shorter OS	Yang et al. (2023)
		Downregulation			
SHMT2	Thyroid cancer	Upregulation	Protein	Advanced TNM stage and shorter PFS	Jin et al. (2021)
	Colorectal cancer	Upregulation	Protein	Advanced TNM stage and lymph node metastasis	Cui et al. (2022a)

DFS, disease-free survival; OS, overall survival; PFS, progression-free survival; SHIN1, SHMT, inhibitor; TNM, tumor, lymph node, metastasis.

A limitation of some anti-metabolite drugs is that multiple targeted metabolic enzymes expressed in both tumor cells and healthy proliferative cells, leading to adverse side-effects and therefore limiting the utilization of anti-metabolite regimens in clinical settings. The protein of MTHFD2 is expressed in immature cells and transformed cells, but importantly not in mature and normal cells. Thus, MTHFD2 is a promising therapeutic target to selectively eradicate tumor cells by disturbing one-carbon metabolism while sparing healthy proliferative cells. LY345899 has been characterized as a potent MTHFD2 inhibitor (Gustafsson et al., 2017). In CRC, the anti-tumor effects of LY345899 on tumor growth and metastasis have been verified *in vitro* and *in vivo* (Ju et al., 2019). In models of castration-resistant prostate cancer, another MTHFD2 inhibitor, DS18561882, combined with enzalutamide could suppress tumor growth (Zhao et al., 2022). DS44960156, a new isozyme-selective MTHFD2 inhibitor, has been developed with higher selectivity for MTHFD2 than MTHFD1 and good ligand efficiency (Kawai et al., 2019) (Table 2).

**TABLE 2:** Targeting one-carbon metabolic enzymes in tumors.

Gene	Methods	Cancer type	Results	Status	Reference
MTHFD2	Inhibitor	Colorectal cancer	Inhibiting growth, lung and peritoneal metastasis	Preclinical	Ju et al. (2019)
	LY345899				
	Inhibitor TH9619	Acute myeloid leukemia	Inhibiting growth	Preclinical	Bonagas et al. (2022)
	Inhibitor DS18561882	Prostate cancer	Inhibiting growth	Preclinical	Zhao et al. (2022)
	siRNA	Non-small-cell lung cancer	Inhibiting growth	Preclinical	Gao et al. (2022)
		Lung adenocarcinoma	Inhibiting growth and metastasis	Preclinical	Shi et al. (2021)
		Lung adenocarcinoma	Inhibiting growth	Preclinical	Mo et al. (2022)
		Ovarian cancer	Inhibiting growth and metastasis	Preclinical	Li et al. (2021b)
		Glioblastoma	Inhibiting growth	Preclinical	Zhu et al. (2022b)
	Administered nanoliposomal siRNA	Prostate cancer	Inhibiting growth	Preclinical	Pällmann et al. (2021)
	shRNA	Acute myeloid leukemia	Inhibiting growth	Preclinical	Pikman et al. (2016)
		Renal cell carcinoma	Inhibiting growth	Preclinical	Green et al. (2019)
		Lung cancer	Inhibiting growth and stemness	Preclinical	Nishimura et al. (2019)
					Chan et al. (2020)
		Pancreatic cancer	Inhibiting growth	Preclinical	Shang et al. (2021)
SHMT1/2	Inhibitor	T-cell lymphoblastic leukemia	Inhibiting growth	Preclinical	Pikman et al. (2022)
	RZ-2994				
	Inhibitor	T-cell lymphoblastic leukemia	Inhibiting growth	Preclinical	Ducker et al. (2017)
	SHIN1				
	Inhibitor	T-cell lymphoblastic leukemia	Inhibiting growth	Preclinical	Garcia-Canaveras et al. (2021)
	SHIN1				

siRNA, small interfering RNA; shRNA, short hairpin RNA; SHIN1, SHMT, inhibitor.

### Targeting SHMTs in cancer

The reversible conversion of serine and tetrahydrofolate to glycine and 5,10- methylene tetrahydrofolates is catalyzed by SHMTs, which is a critical step of one-carbon metabolism. SHMTs have two isoforms, namely, the cytoplasmic SHMT1 and mitochondrial SHMT2. This reaction provides one-carbon units for nucleotide biosynthesis, regulating DNA methylation and NADPH generation. Targeting SHMTs may be exploited as potentially promising therapeutic strategy for developing anti-tumor drugs.

### Targeting SHMT1 in cancer

In some cancers, SHMT1 acts as a tumor promoter. In CRC, SHMT1 functions as a critical metabolic switch, providing one-carbon units between the thymidylate biosynthesis and methionine biosynthesis. Modifying the expression SHMT1 in Apc^min/+^ mice could affect the contributions of purine, thymidylate and methionine biosynthesis, and ultimately determining genome stability to indue CRC initiation (Macfarlane et al., 2011). In non–small cell lung cancer, glycogen synthase kinase 3 (GSK3) mediated the expression of one-carbon metabolic enzymes, especially SHMT1. Nuclear enrichment of GSK3 could suppress expression of SHMT1 in lung cancer cells. Moreover, pharmaceutical inhibition of GSK3 by CHIR99021 confered a metabolic vulnerability to enhance the efficacy of SHMT1/2 inhibitor SHIN1 in lung cancer cells (He et al., 2022). In addition, SHMT1 inhibition induced p53-dependent apoptosis and cell cycle arrest in lung cancer cells (Paone et al., 2014).

SHMT1 functions as a tumor suppressor in hepatocellular carcinoma (HCC) and renal cell carcinoma. In HCC cells, SHMT1 overexpression could impair the metastatic ability of HCCLM3 cells while SHMT1 inhibition could augment the metastasis of HCC cells (Hep3B). Mechanically, SHMT1 inhibition led to increased reactive oxygen species to promote epithelial and mesenchymal transition in HCC cells (Hep3B) (Dou et al., 2019). SHMT1 overexpression significantly retarded the growth of renal cell carcinoma. Homeobox D8 (HOXD8) functions as the upstream regulator of SHMT1. HOXD8 upregulated SHMT1 expression to impair the proliferative and migrative ability of renal cell carcinoma, indicating SHMT1 as a tumor suppressor for renal cell carcinoma (Yang et al., 2023).

### Targeting SHMT2 in cancer

One-carbon units are required for tRNA methylatatin, which is critical for mitochondrial translation and oxidative phosphorylation. In colon cancer, SHMT2 maintained the expression of mitochondrial respiratory chain proteins to sustain oxidative phosphorylation (Morscher et al., 2018). SHMT2 is required for tumor cells for the adaptation to the tumor microenvironment, rendering tumor cells be more sensitive to glycine cleavage system inhibition. SHMT2 could inhibit PKM2 activity and oxidative phosphorylation, conferring survival advantages for tumor cells in poorly vascularized tumor regions (Kim et al., 2015).

The post-translational modifications in the regulation of SHMT2 in CRC cells are under great investigations. SIRT5 could desuccinylate SHMT2 at lysine 280 to enhance its metabolic activity and promote serine catabolism in CRC cells. Hypersuccinylation of SHMT2 at lysine 280 led to impaired enzymatic activity and CRC proliferation (Yang et al., 2018). Lysine acetylation also participates in the regulation of SHMT2 by disrupting its functional structure and inhibiting its enzymatic activity via TRIM21-mediated K63-ubiquitin-lysosome pathway. SHMT2 K95-Ac impaired CRC proliferation *in vivo* and *in vitro* by reducing serine consumption and NADPH generation (Wei et al., 2018). SHMT2 could interact with β-catenin and inhibit the ubiquitylation-mediated degradation of β-catenin, and ultimately promoting CRC proliferation and metastasis. TCF4 could interact with β-catenin to increase SHMT2 expression and form an SHMT2/β-catenin positive feedback loop (Liu et al., 2021b). This SHMT2/β-catenin loop represents a promising therapeutic target for CRC treatment. Herein, targeting SHMT2 represents a potentially attractive strategy for cancer treatment.

SHMT2 also participates in the regulation of 5-fluorouracil chemoresistance of CRC. Chen et al. found that SHMT2 binds cytosolic p53 and prevents cytosolic p53 degradation, leading to impaired autophagy. Under 5-fluorouracil treatment, SHMT2 abrogation could enhance autophagy and inhibit cell apoptosis in CRC (Chen et al., 2021). The p53-SHMT2 interaction may provide novel targets for overcoming chemoresistance. In another study, Pranzini et al. found that 5- fluorouracil -resistant CRC cells exhibit increased reliance to serine by enhanced serine biosynthesis or exogenous serine uptake. The SHMT2-induced serine metabolism represents a metabolic adaptation of 5-fluorouracil-resistant CRC cells to potentiate DNA damage response (Pranzini et al., 2022).

### Clinical implications of SHMT1/2 in cancer

Protein level of SHMT1 has been found to be downregulated in HCC, and reduced SHMT1 expression was associated with decreased overall survival of patients (Dou et al., 2019). SHMT1 protein was reduced in renal cell carcinoma tissues and correlated with poor prognosis of patients. Besides, Shmt1 hemizygosity has been found to be correlated with increased risk for intestinal tumor in Apc^min/+^ mice (Macfarlane et al., 2011).

RZ-2994, a novel inhibitor of SHMT1 and SHMT2, could induce G2 cell cycle arrest in T-cell lymphoblastic leukemia (Pikman et al., 2022). Moreover, RZ-2994 could effectively reduce leukemia burden in the setting of methotrexate resistance. a dual SHMT1/2 inhibitor. Ducker et al. designed a folate-competitive inhibitor of SHMTs, namely, SHIN1. SHIN1 exhibits its potential to specifically target SHMTs and impair cell proliferation in a number of tumor cell lines (Ducker et al., 2017). Another SHMT inhibitor SHIN2 could block the growth of T-cell lymphoblastic leukemia. Moreover, methotrexate-resistant tumor cells exhibited increased sensitivity to SHIN2 (Garcia-Canaveras et al., 2021).

## Conclusion

One-carbon metabolism has been found to be dysregulated in various cancer types due to its role in generation of purine and pyrimidine nucleotides, epigenetic program, and redox homeostasis, and is composed a network of one-carbon metabolic enzymes. MTHFDs and SHMTs are participated in the tumor initiation and progression, gradually being recognized as potential therapeutic anti-tumor targets. Several inhibitors targeting MTHFD2, SHMT1/2 has exerted its potential to decrease tumor burden and inhibit tumor proliferation.
